# Psychological capital, emotional intelligence, and creative teaching behavior among higher education physical education teachers: the mediating role of teacher wellbeing

**DOI:** 10.3389/fpsyg.2026.1860093

**Published:** 2026-07-28

**Authors:** Ruitao Ge

**Affiliations:** Wuhu Youth Sports School, Wuhu, China

**Keywords:** creative teaching behavior, emotional intelligence, physical education, psychological capital, teacher wellbeing

## Abstract

In the dynamic landscape of higher education, Physical Education (PE) teachers face unique pressures that require adaptability and innovation to effectively engage adult learners. Drawing on the Conservation of Resources (COR) theory and the Broaden-and-Build theory, this study investigates the interactive relationships between Psychological Capital (PsyCap), Emotional Intelligence (EI), Wellbeing, and Creative Teaching Behavior (CTB). A cross-sectional survey was conducted with 420 PE teachers from universities across Mainland China. Data were collected using validated self-report instruments, including the Psychological Capital Questionnaire, the Wong and Law Emotional Intelligence Scale, the Teacher Subjective Wellbeing Questionnaire, and a Creative Teaching Behavior Scale. Structural Equation Modeling (SEM) and bias-corrected bootstrapping were employed to analyze the direct and indirect effects among the variables. The results indicate that both PsyCap and EI are significant positive predictors of Creative Teaching Behavior and teacher Wellbeing. Crucially, the analysis indicated significant indirect associations between PsyCap, EI, and Creative Teaching Behavior through Wellbeing, suggesting that Wellbeing may help explain how these personal resources are statistically associated with creative instructional practices. These findings suggest that creative teaching should not be viewed solely as a pedagogical skill, but also as a teaching behavior associated with teachers' psychological resources and wellbeing. The study concludes that higher education institutions should prioritize interventions that enhance teachers‘ psychological resources and emotional regulation to foster wellbeing and sustain creativity in the classroom.

## 1 Introduction

In the landscape of modern higher education, Physical Education (PE) plays a pivotal role not only in promoting physical fitness but also in cultivating lifelong exercise habits and psychological resilience among university students (Zhang and Zhang, 2016). Unlike primary or secondary education, higher education PE requires teachers to possess greater autonomy and adaptability to meet the diverse needs of young adults. Consequently, the traditional command-style teaching is increasingly being replaced by Creative Teaching Behavior, which involves the use of novel, flexible, and original instructional strategies to stimulate student interest and engagement (Beghetto et al., 2014; Xiong et al., 2020). However, implementing creative teaching is demanding; it requires teachers to invest significant cognitive and emotional energy (Huang et al., 2019). Given that PE teachers often face high levels of occupational stress and emotional labor (Yin et al., 2019; Schnitzius et al., 2019), understanding the psychological factors associated with their creative teaching behavior is critical.

To understand how PE teachers maintain the capacity for creativity amidst these demands, the Conservation of Resources (COR) theory (Hobfoll, 1989, 2002) provides a robust framework. COR theory posits that individuals strive to acquire, retain, and foster resources, and that those with abundant resources are less vulnerable to resource loss and more capable of resource gain (Hobfoll et al., 2018). In the context of teaching, personal resources such as Psychological Capital (PsyCap) and Emotional Intelligence (EI) serve as reservoirs of energy. According to the “gain spiral” hypothesis of COR theory (Salanova et al., 2010), these positive psychological resources can lead to enhanced wellbeing, which in turn fuels positive work behaviors like creative teaching.

Psychological Capital, defined as a positive psychological state characterized by self-efficacy, optimism, hope, and resilience (Luthans et al., 2007), has been identified as a critical predictor of teacher performance. Recent studies indicate that PsyCap acts as a protective factor against burnout and facilitates positive outcomes such as professional commitment (Zhang et al., 2025; Bertieaux et al., 2024). Specifically in the context of physical education, Deng et al. (2020) found that resilience, a core component of PsyCap, significantly mediates the relationship between personality traits and creative teaching.

Parallel to PsyCap, Emotional Intelligence (EI)—the ability to perceive, use, understand, and manage emotions (Salovey and Mayer, 1990)—is essential for PE teachers who engage in high-intensity interactions with students. Su et al. (2022) demonstrated that teachers with high EI are better equipped to maintain work engagement, which subsequently promotes teaching for creativity. Since PE teaching involves dynamic environments and constant social interaction, EI allows teachers to navigate emotional challenges effectively, preserving the cognitive space required for creativity (Corcoran and Tormey, 2013).

While PsyCap and EI provide the potential for creativity, Wellbeing represents the optimal psychological state that may translate these potentials into action. According to Fredrickson's (2001) Broaden-and-Build theory, positive emotions and wellbeing expand an individual's thought-action repertoire, encouraging novel and exploratory behaviors. Empirical evidence supports this link; for instance, Zhang et al. (2025) found that wellbeing mediates the effect of PsyCap on professional commitment, suggesting that psychological resources function by first enhancing the teacher's subjective wellness. Similarly, Bertieaux et al. (2024) highlighted that teacher wellbeing is not merely an outcome but a precursor to effective teaching behaviors. It is plausible to hypothesize that PE teachers with high PsyCap and EI experience greater wellbeing, which then provides the motivational impetus for creative teaching behavior.

Despite growing interest in teacher psychology and creative teaching, several important gaps remain. First, previous studies have often examined Psychological Capital or Emotional Intelligence separately, leaving limited understanding of how these two personal resources are jointly associated with Creative Teaching Behavior. Second, much of the existing research has focused on general teacher populations or K−12 educational settings, whereas higher education PE teachers face distinctive professional demands, including adult student engagement, physical safety concerns, performance-based instruction, and institutional expectations for teaching and research. Third, although teacher wellbeing has been widely discussed as an important outcome of psychological resources, its potential role as a statistical mediator linking personal resources to creative teaching behavior remains underexplored, particularly in the context of higher education physical education.

To address these gaps, the present study examines the relationships among Psychological Capital, Emotional Intelligence, teacher wellbeing, and Creative Teaching Behavior among higher education PE teachers in Mainland China. Guided by Conservation of Resources theory and Broaden-and-Build theory, the central research question is whether teacher wellbeing statistically mediates the associations between teachers' personal psychological resources, namely Psychological Capital and Emotional Intelligence, and their self-reported Creative Teaching Behavior. Thus, the primary focus of this study is not merely to identify direct relationships among these variables, but to test an indirect association model in which wellbeing may help explain how personal psychological resources are associated with creative teaching behavior.

## 2 Literature review

### 2.1 Creative teaching behavior in physical education

In the evolving landscape of higher education, Creative Teaching Behavior (CTB) has emerged as a critical pedagogical competency rather than a mere instructional embellishment. Defined as the generation and implementation of novel, appropriate, and flexible instructional approaches (Beghetto and Kaufman, 2014; Xiong et al., 2020), CTB in physical education involves adapting to dynamic sporting environments, diverse student skill levels, and the dual mandate of physical and psychological development. Unlike standardized instruction, creative teaching requires teachers to act as “adaptive experts,” capable of improvising solutions to engage students in lifelong physical activity (Sawyer, 2011; Zhang and Zhang, 2016).

However, the enactment of creativity is resource-intensive. It demands emotional stability, cognitive flexibility, and risk-taking behaviors (Huang et al., 2019). Within the specific context of PE, teachers face unique stressors, including safety concerns, large class sizes, and the public nature of performance, which can inhibit creative expression (Deng et al., 2020). While existing literature has extensively documented the outcomes of creative teaching on student motivation, less attention has been paid to the antecedents—specifically, the internal psychological reservoirs that enable PE teachers to sustain such demanding behaviors.

### 2.2 Psychological capital: the HERO within teachers

To understand the psychological resources necessary for creative teaching, scholars increasingly turn to Positive Organizational Behavior (POB). Central to this is Psychological Capital (PsyCap), defined by Luthans et al. (2007) as a higher-order core construct comprising four state-like resources: hope, efficacy, resilience, and optimism (HERO).

Self-Efficacy refers to the conviction in one's ability to mobilize cognitive resources to execute specific tasks (Bandura, 1997; Luthans and Youssef-Morgan, 2017). In teaching, efficacy is a robust predictor of instructional quality and persistence (Skaalvik and Skaalvik, 2016).

Hope involves the agency to set goals and the pathways to achieve them (Snyder et al., 1991).

Optimism is the generalized expectation of positive outcomes and a positive attributional style (Seligman, 1998).

Resilience represents the capacity to bounce back from adversity or failure (Masten, 2001), which is particularly crucial for teachers facing the “praxis shock” of classroom management (Hong, 2012).

A recent scoping review by Bertieaux et al. (2024) analyzed 32 empirical studies and confirmed a consistent positive correlation between PsyCap and desirable work outcomes, such as job satisfaction and performance. Furthermore, PsyCap serves as a buffer against negative outcomes like stress, anxiety, and burnout (Avey et al., 2011; Min et al., 2015; Ferradás et al., 2019). In the context of education, teachers with high PsyCap are better equipped to navigate the emotional labor of teaching (Carmona-Halty et al., 2021) and are more likely to exhibit innovative behaviors because they view challenges as opportunities rather than threats (Lupşa et al., 2020; Datu and Valdez, 2019). For Higher Education PE teachers, who must balance academic research pressures with physical instruction, PsyCap acts as a critical personal resource reservoir (Grover et al., 2018).

### 2.3 Emotional intelligence as a relational resource

Parallel to the cognitive-motivational resources of PsyCap, Emotional Intelligence (EI) provides the necessary affective capability for effective teaching. EI is conceptualized as the ability to perceive, understand, use, and manage emotions in oneself and others (Mayer and Salovey, 1997). Teaching is inherently an emotional practice (Hargreaves, 1998), and PE teaching, with its high frequency of social interaction and physical vulnerability, requires heightened emotional regulation (Yin et al., 2019).

Research suggests that EI is antecedent to effective classroom management and teacher-student relationships (Corcoran and Tormey, 2013). Teachers with high EI are better at diagnosing student boredom or anxiety and adjusting their teaching strategies creatively to re-engage learners (Su et al., 2022). Furthermore, EI helps prevent the depletion of resources. According to the Conservation of Resources (COR) theory (Hobfoll, 1989), individuals strive to protect their resources. Teachers with low EI may exhaust their energy regulating negative emotions, leaving little cognitive space for creativity. Conversely, high-EI teachers can effectively manage the “emotional labor” of the profession, preserving their energy for creative endeavors (Mérida-López and Extremera, 2017).

### 2.4 Teacher wellbeing: beyond the absence of stress

While PsyCap and EI are capacities, wellbeing is the optimal psychological state that fuels performance. The conceptualization of wellbeing has evolved from a hedonic perspective (focusing on subjective happiness and life satisfaction) (Diener, 1984) to a eudaimonic perspective (focusing on meaning, self-realization, and functioning) (Ryff, 1989; Waterman, 1993). In the workplace, this is often synthesized into “Occupational Wellbeing,” which includes affective, motivational, and cognitive dimensions (Van Horn et al., 2004).

Bertieaux et al. (2024) highlight a critical gap: while teacher burnout is well-studied (Maslach et al., 2001; Madigan et al., 2023), positive teacher wellbeing remains under-explored as an independent construct. This is a significant oversight because the absence of ill-being does not equate to the presence of wellbeing (Keyes, 2002).

Empirical evidence strongly links psychological resources to wellbeing. PsyCap has been found to be a strong predictor of subjective and psychological wellbeing across various cultures and professions (Avey et al., 2011; Luthans et al., 2013; Siu et al., 2015). For teachers specifically, PsyCap fosters a sense of thriving and reduces the intention to quit (Hong, 2012; Arnup and Bowles, 2016). When teachers possess high PsyCap and EI, they are more likely to experience positive emotions (e.g., flow, engagement), which constitutes a high level of wellbeing (Bakker and Oerlemans, 2011).

### 2.5 Theoretical framework and hypotheses development

The mechanism linking wellbeing to creative teaching can be explained by Fredrickson's (2001); Fredrickson (2004) Broaden-and-Build theory. This theory posits that positive emotions (a core component of wellbeing) broaden an individual's momentary thought-action repertoire. Unlike negative emotions, which narrow focus for survival (fight or flight), positive emotions encourage exploration, play, and novel thinking.

For a PE teacher, a state of wellbeing serves as a cognitive expansion mechanism. When a teacher feels psychologically well, they are more open to experimenting with new drills, adopting flexible assessment methods, and engaging in creative problem-solving in the classroom (Caprara et al., 2006). Conversely, teachers experiencing low wellbeing or burnout are likely to retreat to rigid, command-style teaching methods to conserve energy (Shen et al., 2015). Therefore, wellbeing is not just an outcome of resources, but a proximal precursor to creative behavior.

Hobfoll's (1989, 2002) Conservation of Resources (COR) theory provides the overarching framework for integrating PsyCap, EI, Wellbeing, and Creative Teaching. The theory's core tenet is that individuals invest resources to protect against loss and gain new resources. A key corollary is the “gain spiral” hypothesis (Salanova et al., 2010), which suggests that resources breed resources.

In this study's context, PsyCap and EI act as primary personal resources. Teachers invest these resources to navigate the demands of higher education PE. Successful investment leads to resource gain in the form of psychological wellbeing. This enhanced state of wellbeing then acts as an energetic reservoir that supports the high-cost behavior of Creative Teaching.

PsyCap, EI, and Wellbeing: high PsyCap teachers (hopeful, resilient) and High EI teachers (emotionally regulated) perceive job demands as manageable challenges rather than threats (Lazarus and Folkman, 1984). This cognitive appraisal reduces stress and directly enhances wellbeing (referencing Bertieaux et al., 2024; Braunstein-Bercovitz et al., 2012).

The mediating role of wellbeing: while PsyCap and EI provide the capacity to be creative, wellbeing provides the impetus. Recent studies in organizational psychology suggest that personal resources influence performance outcomes primarily through their impact on the individual's psychological state (Grover et al., 2018; Adil and Kamal, 2016). For instance, Zhang et al. (2025) and Singhal and Rastogi (2018) found that subjective wellbeing mediates the relationship between PsyCap and commitment. Extending this logic to performance, it is hypothesized that wellbeing mediates the relationship between personal resources (PsyCap, EI) and Creative Teaching Behavior.

Beyond the conceptual relevance of Psychological Capital, Emotional Intelligence, teacher wellbeing, and Creative Teaching Behavior, prior empirical studies provide support for the expected associations among these constructs. Psychological Capital has been consistently linked to positive teacher and employee outcomes, including job satisfaction, work engagement, organizational commitment, and performance. Avey et al. (2011), for example, reported that PsyCap was positively associated with desirable work attitudes and behaviors and negatively associated with stress and burnout. In educational contexts, PsyCap has also been found to support teachers' adaptive functioning and professional commitment, suggesting that teachers with higher levels of hope, efficacy, resilience, and optimism may be better positioned to cope with occupational demands and maintain positive work-related states.

Empirical research also supports the association between Emotional Intelligence and teacher functioning. Emotional Intelligence has been linked to teachers' emotional regulation, classroom management, work engagement, and wellbeing. For example, Mérida-López and Extremera (2017) showed that EI is closely related to teacher burnout and emotional functioning, while Vesely et al. (2013) highlighted the contribution of EI to teacher efficacy and wellbeing. In the context of teaching for creativity, Su et al. (2022) found that teachers' Emotional Intelligence was associated with teaching for creativity through work engagement, indicating that teachers' ability to perceive, understand, and regulate emotions may be relevant to creativity-supportive instructional behavior.

The relationship between wellbeing and creative teaching is also supported by previous research. Teachers with higher wellbeing are more likely to experience positive emotions, engagement, and a sense of professional efficacy, which may be associated with openness, flexibility, and willingness to experiment with new instructional strategies. This is consistent with Broaden-and-Build theory, which suggests that positive emotional states broaden individuals' thought-action repertoires and facilitate exploratory and creative behaviors. In physical education, where teaching often requires improvisation, adaptation, and immediate responses to students' physical and emotional needs, wellbeing may be particularly relevant to teachers' creative instructional practices.

Taken together, these empirical findings suggest that Psychological Capital and Emotional Intelligence may be associated with Creative Teaching Behavior both directly and indirectly through teacher wellbeing. PsyCap and EI represent personal psychological and emotional resources that may support teachers' positive functioning, while wellbeing may represent a proximal psychological state associated with the enactment of creative teaching behavior. Therefore, the present study extends previous research by testing an integrated indirect association model in which teacher wellbeing statistically mediates the relationships between PsyCap, EI, and Creative Teaching Behavior among higher education PE teachers.

Based on the theoretical integration of COR and Broaden-and-Build theories, along with the empirical evidence reviewed above, we postulate that personal psychological resources (PsyCap and EI) serve as antecedents to teacher wellbeing, which in turn fosters creative teaching behavior. Consequently, the following hypotheses are proposed:

H1: psychological capital is positively associated with teacher wellbeing among higher education PE teachers.
H2: emotional intelligence is positively associated with teacher wellbeing among higher education PE teachers.
H3: psychological capital is positively associated with creative teaching behavior among higher education PE teachers.
H4: emotional intelligence is positively associated with creative teaching behavior among higher education PE teachers.
H5: teacher wellbeing is positively associated with creative teaching behavior among higher education PE teachers.
H6: teacher wellbeing statistically mediates the association between psychological capital and creative teaching behavior among higher education PE teachers.
H7: teacher wellbeing statistically mediates the association between emotional intelligence and creative teaching behavior among higher education PE teachers.

**Figure 1:**
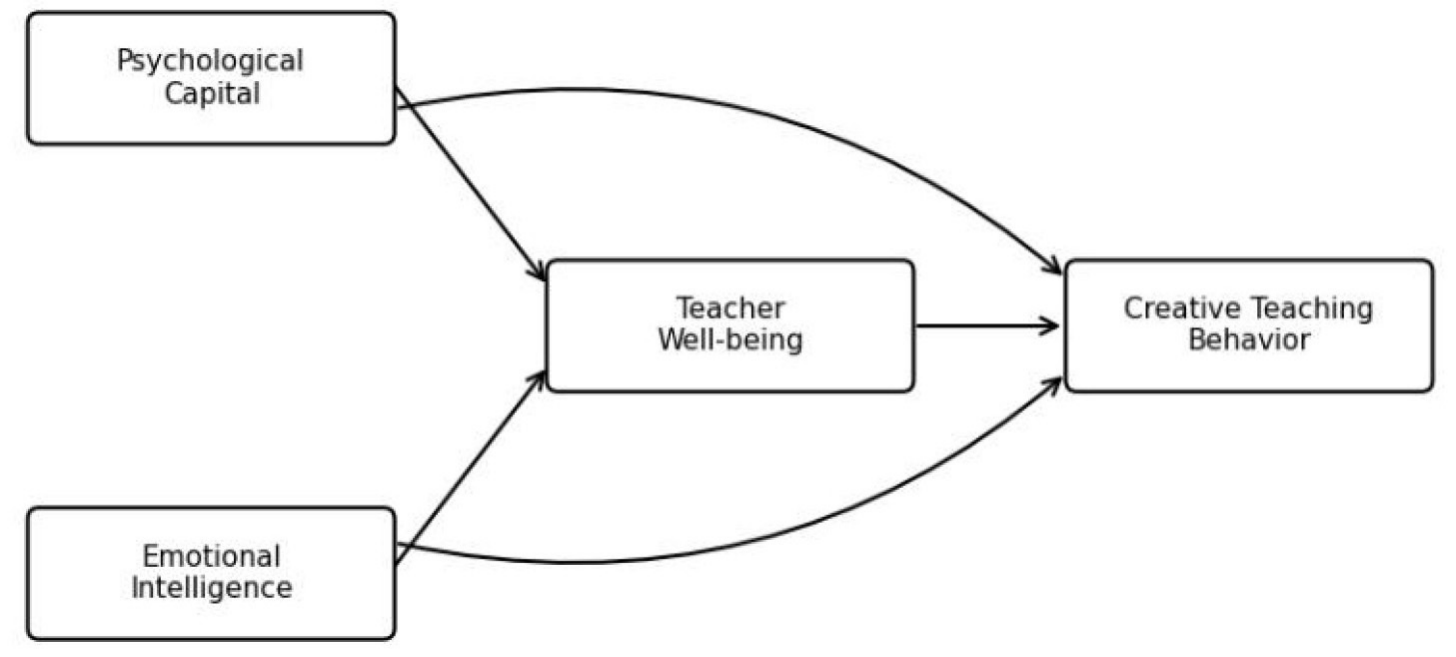
Conceptual model of the hypothesized relationships among psychological capital, emotional intelligence, teacher wellbeing, and creative teaching behavior.

Based on the theoretical framework and hypotheses developed above, Figure 1 presents the conceptual model of the study. Psychological Capital and Emotional Intelligence are specified as personal psychological resources, teacher wellbeing is specified as a mediating variable, and Creative Teaching Behavior is specified as the outcome variable. The model proposes that Psychological Capital and Emotional Intelligence are positively associated with Creative Teaching Behavior both directly and indirectly through teacher wellbeing.

## 3 Method

### 3.1 Participants and procedure

This study adopted a cross-sectional survey design to examine the hypothesized relationships. The target population comprised physical education (PE) teachers currently employed in higher education institutions (universities and colleges) across Mainland China. Data collection was conducted from September to November 2025 using an online survey platform (i.e., Wenjuanxing). Convenience sampling was employed, supplemented by snowball sampling techniques, where initial participants were encouraged to invite eligible colleagues to participate.

To ensure the quality of the data, specific inclusion criteria were set: participants must be full-time PE teachers in higher education institutions. Before starting the survey, all participants were presented with an informed consent form detailing the study's purpose, the anonymity of their responses, and their right to withdraw at any time.

A total of 550 questionnaires were initially distributed. Following the data cleaning procedures recommended by Podsakoff et al. (2003), responses were screened for validity. Questionnaires were excluded if they: (1) were completed in < 120 s, indicating insufficient attention; (2) showed straight-lining patterns (e.g., selecting the same option for all items); or (3) had significant missing data. Consequently, 420 valid questionnaires were retained for the final analysis, yielding an effective response rate of around 76%. This sample size exceeds the recommended minimum of 200 for Structural Equation Modeling (SEM) and satisfies the item-to-respondent ratio suggested by Kline (2015).

The final sample consisted of 255 males (60.7%) and 165 females (39.3%). The average age of the participants was approximately 37.8 years (SD = 7.5). Regarding educational background, around 60% held a master's degree or higher, which reflects the typical qualification requirements in higher education. In terms of academic rank, about 18% were Teaching Assistants, 42% were Lecturers, 28% were Associate Professors, and 12% were Professors. The distribution of teaching experience showed that 35% had taught for < 5 years, 38% for 6–15 years, and 27% for more than 15 years.

Convenience sampling supplemented by snowball sampling was considered appropriate for the present study for several reasons. First, higher education physical education teachers represent a specialized professional population that is geographically dispersed across universities in Mainland China, and no comprehensive national sampling frame is available. Consequently, probability-based sampling was not practically feasible. Second, the primary objective of this study was to examine the theoretically hypothesized relationships among latent psychological constructs rather than to estimate population prevalence or generate nationally representative estimates. Structural equation modeling is commonly applied in theory-testing research using non-probability samples, provided that the sample size is adequate and the measurement model demonstrates satisfactory reliability and validity. The final sample of 420 participants exceeded commonly recommended sample sizes for SEM and provided sufficient statistical power for the hypothesized model. Nevertheless, the use of convenience sampling may introduce selection bias and limit the generalizability of the findings. Therefore, the results should be interpreted with appropriate caution.

### 3.2 Measures

All scales used in this study were derived from widely cited, validated instruments in existing literature (See Appendix). All measurement instruments in this study were administered in Chinese. To ensure linguistic equivalence and cultural appropriateness, a standardized translation and back-translation procedure was employed following established guidelines in cross-cultural research (Brislin, 1980).

First, the original English versions of all scales were independently translated into Chinese by two bilingual researchers with doctoral-level training in psychology and education. The two translated versions were then compared and reconciled through discussion to produce a preliminary Chinese version. Second, this Chinese version was back-translated into English by an independent bilingual expert who was not involved in the initial translation and was blind to the original instruments. The back-translated version was subsequently compared with the original English scales to identify and resolve any discrepancies in meaning or nuance.

To further enhance content validity and contextual relevance, the final Chinese versions were reviewed by two experts in physical education pedagogy and educational psychology. Minor wording adjustments were made to ensure clarity and suitability for higher education physical education teachers in Mainland China, without altering the original conceptual meanings of the items. A pilot test with a small group of PE teachers (*N* = 30) confirmed that all items were clear and comprehensible.

#### 3.2.1 Psychological capital

Psychological Capital was measured using the Psychological Capital Questionnaire developed by Luthans et al. (2007). This scale is considered the “gold standard” in Positive Organizational Behavior research. It comprises 24 items across four dimensions: self-efficacy, hope, resilience, and optimism. A sample item is “I feel confident analyzing a long-term problem to find a solution.” The scale has demonstrated excellent psychometric properties across diverse cultural contexts (Avey et al., 2011). Items were measured on a 6-point Likert scale (1 = strongly disagree to 6 = strongly agree).

#### 3.2.2 Emotional intelligence

Emotional Intelligence was assessed using the Wong and Law Emotional Intelligence Scale (WLEIS), originally developed by Wong and Law (2002) and validated for a Chinese sample by Kong (2017). This scale is widely recognized for its robust validity in organizational settings, particularly in the Asian context. It consists of 16 items measuring four dimensions: self-emotion appraisal, others' emotion appraisal, use of emotion, and regulation of emotion. A sample item includes “I have a good sense of why I have certain feelings.”

#### 3.2.3 Teacher wellbeing

To assess teacher wellbeing, we employed the Teacher Subjective Wellbeing Questionnaire (TSWQ) developed by Renshaw et al. (2015).

The TSWQ is a distinct measure designed to assess teachers' optimal functioning. It consists of 8 items encompassing two subscales: teaching efficacy and school connectedness. A sample item is “I feel like I belong at this school.” This instrument aligns with the eudaimonic perspective of wellbeing widely discussed in positive psychology (Diener et al., 2010; Ryff, 1989).

#### 3.2.4 Creative teaching behavior

Creative Teaching Behavior was measured using the Creative Teaching Behavior Scale developed by Rubenstein et al. (2013). This scale was designed to assess teachers' perceptions of the behaviors and practices that foster creativity in the classroom. It includes items that evaluate how teachers incorporate creative teaching strategies, encourage innovation in their students, and adapt their instructional approaches to promote creative thinking. The scale focuses on various dimensions such as openness to new ideas, encouragement of divergent thinking, autonomy in learning, and problem-solving and innovation. These items reflect teachers' creative behaviors and their approach to fostering an environment that nurtures creativity in the classroom. Responses were recorded on a Likert-type scale, with higher scores indicating a stronger presence of creative teaching behaviors.

#### 3.2.5 Measurement modeling strategy

Prior to testing the structural relationships, a two-step approach to structural equation modeling was adopted, as recommended by Anderson and Gerbing (1988). In the first step, confirmatory factor analyses (CFAs) were conducted to examine the adequacy of the measurement model. In the second step, the hypothesized structural model was tested based on the validated measurement model.

All constructs were modeled as latent variables using their respective observed indicators. Psychological Capital (PsyCap) was specified as a second-order latent construct composed of four first-order dimensions: self-efficacy, hope, resilience, and optimism, consistent with its theoretical conceptualization (Luthans et al., 2007). Emotional Intelligence, Teacher Wellbeing, and Creative Teaching Behavior were modeled as first-order latent constructs, with all items loading on their respective factors.

Item parceling was not applied in the present study. Although parceling can simplify model estimation, all individual items were retained as indicators in order to preserve the theoretical integrity of each construct and allow for a more stringent test of construct validity. This approach is particularly appropriate for theory-driven models involving multidimensional psychological constructs.

The measurement model was estimated using the maximum likelihood (ML) method in AMOS 24.0. Before conducting the structural equation modeling analyses, the assumptions of normality, linearity, and multicollinearity were examined. Normality was assessed using skewness and kurtosis values for the observed variables. All skewness and kurtosis values were within the commonly accepted thresholds of |skewness| < 2 and |kurtosis| < 7, indicating no severe violation of normality. Linearity was examined through scatterplots among the main study variables, which showed generally linear patterns. Multicollinearity was assessed using tolerance and variance inflation factor values. All tolerance values were above 0.10 and all VIF values were below 5.00, suggesting that multicollinearity was not a serious concern. Therefore, the data were considered suitable for subsequent SEM analyses.

Convergent validity was assessed by examining standardized factor loadings, composite reliability (CR), and average variance extracted (AVE). Factor loadings >0.50, CR values above 0.70, and AVE values exceeding 0.50 were considered indicative of adequate convergent validity (Fornell and Larcker, 1981). Discriminant validity was evaluated by comparing the square root of AVE for each construct with its correlations with other constructs.

### 3.3 Data analysis

Data analysis was performed using SPSS 26.0 and AMOS 24.0. Demographic variables, including gender, age, highest educational qualification, teaching experience, and academic rank, were collected to describe the sample characteristics. However, these variables were not included as control variables in the main structural model. This decision was made because the present study was primarily theory-driven and focused on the relationships among Psychological Capital, Emotional Intelligence, teacher wellbeing, and Creative Teaching Behavior, rather than on demographic prediction. In addition, the demographic variables were not central to the theoretical framework derived from Conservation of Resources theory and Broaden-and-Build theory. Therefore, to preserve model parsimony and avoid over-controlling for variables outside the core theoretical model, the structural analysis focused on the hypothesized psychological constructs.

Differences in response formats do not affect SEM estimation based on covariance structures; all latent constructs were modeled using their original item metrics. We calculated descriptive statistics and Pearson correlation coefficients. Because all variables were measured using self-report questionnaires administered at a single time point, common method variance was a potential concern. Harman's single-factor test was conducted as a preliminary diagnostic procedure (Podsakoff et al., 2003). However, this test has recognized limitations and cannot rule out common method bias. Therefore, the results were interpreted cautiously, and common method variance was explicitly acknowledged as a limitation of the study. Confirmatory Factor Analysis (CFA) was employed to verify the construct validity. Model fit was evaluated using multiple fit indices, including the Comparative Fit Index (CFI), Tucker–Lewis Index (TLI), Root Mean Square Error of Approximation (RMSEA), and Standardized Root Mean Square Residual (SRMR). Following commonly used SEM guidelines, CFI and TLI values around or above 0.90, RMSEA values around or below 0.08, and SRMR values around or below 0.08 were treated as indicative of acceptable model fit. These values were interpreted as general benchmarks rather than rigid cut-off rules, and model adequacy was evaluated based on the overall pattern of fit indices and theoretical plausibility. Structural Equation Modeling (SEM) was used to test the direct relationships. To test the mediating effect of Wellbeing, we utilized the bias-corrected bootstrapping method with 5,000 resamples, as recommended by Preacher and Hayes (2008). This method is widely accepted for its high statistical power in detecting indirect effects without assuming a normal distribution.

## 4 Results

### 4.1 Descriptive statistics

The descriptive statistics for the key variables in this study are summarized in Table 1. The sample included 420 valid responses from Physical Education (PE) teachers at higher education institutions. The mean scores for the four primary constructs—Psychological Capital (PsyCap), Emotional Intelligence (EI), Wellbeing, and Creative Teaching Behavior (CTB)—indicate that, on average, the respondents exhibit relatively high levels of these psychological resources and teaching behaviors.

**Table 1:** Descriptive statistics for the key variables.

Variable	Mean (M)	Standard deviation (SD)	Range
Psychological capital (PsyCap)	4.52	0.68	1.00–6.00
Emotional intelligence (EI)	4.30	0.60	1.00–6.00
Wellbeing	3.88	0.73	1.00–4.00
Creative teaching behavior (CTB)	4.60	0.75	1.00–7.00

The mean values suggest that PE teachers generally report strong psychological resources, emotional intelligence, and engagement in creative teaching behaviors. The standard deviations are moderate, indicating some variability within the sample, which is typical in psychological and behavioral data. These findings indicate a relatively positive outlook on the emotional and psychological resources available to PE teachers in the higher education context.

### 4.2 Correlation analysis

A Pearson correlation analysis was conducted to examine the relationships between the key variables: psychological capital, emotional intelligence, wellbeing, and creative teaching behavior. The results are shown in Table 2.

**Table 2:** Pearson correlations among the main study variables.

Variable	PsyCap	EI	Wellbeing	CTB
Psychological capital (PsyCap)	1	0.63^**^	0.57^**^	0.52^**^
Emotional intelligence (EI)	0.63^**^	1	0.61^**^	0.56^**^
Wellbeing	0.57^**^	0.61^**^	1	0.66^**^
Creative teaching behavior (CTB)	0.52^**^	0.56^**^	0.66^**^	1

^**^p < 0.05.

The results reveal that all variables are positively correlated. Particularly noteworthy is the strong positive relationship between Emotional Intelligence and Wellbeing (*r* = 0.61, *p* < 0.01), and between Wellbeing and Creative Teaching Behavior (*r* = 0.66, *p* < 0.01). These results show that Emotional Intelligence was positively correlated with both Wellbeing and Creative Teaching Behavior. The observed correlations were consistent with the hypothesized positive associations among the study variables.

### 4.3 Reliability, validity, and model fit

Before testing the structural relationships, the reliability, convergent validity, discriminant validity, and overall fit of the measurement model were examined. As shown in Table 3, all constructs demonstrated satisfactory internal consistency. Cronbach's alpha values ranged from 0.85 to 0.95, exceeding the recommended threshold of 0.70. Composite reliability values ranged from 0.86 to 0.95, also meeting the recommended criterion of 0.70. The standardized factor loadings ranged from 0.60 to 0.88 and were all statistically significant at *p* < 0.001, indicating that the observed indicators loaded adequately on their corresponding latent constructs. The AVE values ranged from 0.51 to 0.60 at the dimension level and from 0.54 to 0.59 at the construct level, meeting the recommended threshold of 0.50. These results support the convergent validity of the measurement model.

**Table 3:** Reliability and convergent validity of the measurement model.

Construct/Dimension	Number of items	Standardized factor loadings	Cronbach's α	CR	AVE
Psychological capital	24	0.60–0.84	0.94	0.93	0.54
Self-efficacy	6	0.69–0.84	0.89	0.90	0.60
Hope	6	0.67–0.82	0.88	0.89	0.58
Resilience	6	0.60–0.78	0.85	0.86	0.51
Optimism	6	0.62–0.80	0.86	0.87	0.53
Emotional intelligence	16	0.66–0.86	0.93	0.94	0.58
Teacher wellbeing	8	0.68–0.86	0.90	0.91	0.56
Creative teaching behavior	13	0.69–0.88	0.95	0.95	0.59

CR, composite reliability; AVE, average variance extracted. Psychological Capital was modeled as a second-order construct consisting of self-efficacy, hope, resilience, and optimism. All standardized factor loadings were statistically significant at *p* < 0.001.

Discriminant validity was assessed using the Fornell–Larcker criterion. As presented in Table 4, the square root of AVE for each latent construct was greater than its correlations with the other constructs. Specifically, the square roots of AVE were 0.73 for Psychological Capital, 0.76 for Emotional Intelligence, 0.75 for Teacher Wellbeing, and 0.77 for Creative Teaching Behavior, all of which exceeded the corresponding inter-construct correlations. These results indicate adequate discriminant validity among the four constructs.

**Table 4:** Discriminant validity of the latent constructs.

Construct	1	2	3	4
1. Psychological capital	0.73			
2. Emotional intelligence	0.63	0.76		
3. Teacher wellbeing	0.57	0.61	0.75	
4. Creative teaching behavior	0.52	0.56	0.66	0.77

Diagonal values represent the square root of AVE. Off-diagonal values represent correlations among the latent constructs. Discriminant validity is supported when the square root of AVE for each construct exceeds its correlations with other constructs.

The overall goodness-of-fit indices further supported the adequacy of both the measurement and structural models. As shown in Table 5, the χ^2^/df ratios for the measurement model, χ^2^/df = 1.98, and the structural model, χ^2^/df = 2.19, were below the recommended threshold of 3.00. Incremental fit indices also supported model adequacy, with CFI values of 0.93 and 0.94 and TLI values of 0.92 for the measurement and structural models, respectively. Absolute fit was also satisfactory, as reflected by RMSEA values of 0.048 and 0.058 and SRMR values of 0.046 and 0.052. Taken together, the reliability, validity, and model fit results indicate that the measurement model was adequate for subsequent structural analysis.

**Table 5:** Model fit indices for the measurement and structural models.

Fit index	Measurement model (CFA)	Structural model (SEM)	Cut-off criteria
χ^2^	1986.40	2215.10	—
df	1004	1012	—
χ^2^/df	1.98	2.19	< 3.00 (Kline, 2015)
CFI	0.93	0.94	≥0.90 (acceptable); ≥0.95 (good)
TLI	0.92	0.92	≥0.90 (acceptable); ≥0.95 (good)
RMSEA	0.048	0.058	≤0.08 (acceptable); ≤ 0.06 (good)
SRMR	0.046	0.052	≤ 0.08 (Hu and Bentler, 1999)

CFI, Comparative Fit Index; TLI, Tucker–Lewis Index; RMSEA, Root Mean Square Error of Approximation; SRMR, Standardized Root Mean Square Residual. Cut-off criteria were based on Hu and Bentler (1999) and Kline (2015).

### 4.4 Direct effects

The next step in the analysis was to examine the direct effects of the personal psychological resources (PsyCap and EI) on Creative Teaching Behavior (CTB) and Wellbeing. The path coefficients for these direct effects are provided in Table 6.

**Table 6:** Direct effects among variables.

Path	β (Path coefficient)	Standard error (SE)	*p*-value
Psychological capital → creative teaching behavior	0.21^**^	0.06	0.002
Emotional intelligence → creative teaching behavior	0.31^**^	0.05	0.001
Psychological capital → wellbeing	0.36^**^	0.07	0.001
Emotional intelligence → wellbeing	0.45^**^	0.06	0.001

^**^p < 0.05.

Both Psychological Capital and Emotional Intelligence were found to have significant positive effects on Creative Teaching Behavior and Wellbeing. Specifically, Emotional Intelligence showed a stronger effect on both Creative Teaching Behavior (β = 0.31, *p* < 0.01) and Wellbeing (β = 0.45, *p* < 0.01). This suggests that PE teachers with higher levels of emotional intelligence are more likely to engage in creative teaching practices and report higher levels of wellbeing.

### 4.5 Mediating role of wellbeing

Next, the mediating effect of Wellbeing was tested using the bias-corrected bootstrapping method (Preacher and Hayes, 2008). The indirect effects, along with their 95% confidence intervals, are shown in Table 7. The bootstrapping analysis revealed significant indirect effects through Wellbeing in the associations between Psychological Capital and Creative Teaching Behavior, as well as between Emotional Intelligence and Creative Teaching Behavior.

**Table 7:** Mediating effect of wellbeing.

Path	Indirect effect (β)	Standard error (SE)	95% Confidence interval
Psychological capital → wellbeing → creative teaching behavior	0.18^**^	0.05	[0.09, 0.29]
Emotional intelligence → wellbeing → creative teaching behavior	0.23^**^	0.04	[0.15, 0.35]

^**^p < 0.05.

The bootstrapping results indicated that Wellbeing showed a significant statistical mediating role in the associations between Psychological Capital, Emotional Intelligence, and Creative Teaching Behavior. Specifically, the indirect effect of Psychological Capital on Creative Teaching Behavior through Wellbeing (β = 0.18, *p* < 0.01) and the indirect effect of Emotional Intelligence on Creative Teaching Behavior through Wellbeing (β = 0.23, *p* < 0.01) were both significant, suggesting that teachers with higher psychological resources tended to report higher wellbeing and greater creative teaching behavior.

**Figure 2:**
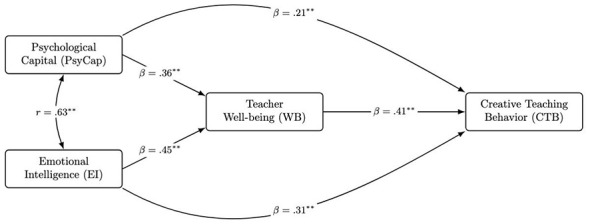
Structural equation model of the relationships among psychological capital, emotional intelligence, wellbeing, and creative teaching behavior.

Figure 2 illustrates the hypothesized and tested structural model. Psychological capital and emotional intelligence were specified as exogenous latent variables, teacher wellbeing as a mediating variable, and creative teaching behavior as the outcome variable. Standardized path coefficients are reported. All displayed paths were statistically significant (*p* < 0.01).

## 5 Discussion

The primary purpose of this study was to construct and test an integrative model linking Psychological Capital (PsyCap), Emotional Intelligence (EI), Wellbeing, and Creative Teaching Behavior (CTB) among Higher Education Physical Education (PE) teachers. The structural equation modeling results largely supported the hypothesized associations, suggesting that PsyCap and EI were positively related to creative teaching behavior and that teacher wellbeing showed a significant statistical mediating role in these associations. These findings extend the application of the Conservation of Resources (COR) theory (Hobfoll, 1989, 2002) and the Broaden-and-Build theory (Fredrickson, 2001) into the specific domain of higher education sports pedagogy.

Our findings indicate that PsyCap was positively associated with Creative Teaching Behavior, suggesting that the composite resources of Hope, Efficacy, Resilience, and Optimism (HERO) may be relevant to instructional innovation. This aligns with Luthans et al.'s (2007) conceptualization of PsyCap as a developmental state that goes beyond human capital (what you know) and social capital (who you know) to “who you are.”

Specifically, the positive link between PsyCap and creativity in PE teachers corroborates Sweetman et al. (2011), who argued that creative performance inherently involves risk-taking and perseverance—traits underpinned by resilience and hope. In the context of physical education, where teachers often face the “praxis shock” of managing large classes and safety concerns (Hong, 2012), PsyCap may serve as a psychological resource associated with better adaptation. As noted by Avey et al. (2011) in their meta-analysis, PsyCap is associated with lower burnout and higher citizenship behaviors; our study adds to this literature by showing that PsyCap was also positively associated with self-reported creative teaching behavior. Furthermore, the role of self-efficacy, as a component of PsyCap, is consistent with Bandura's (1997) social cognitive theory and Skaalvik and Skaalvik's (2016) findings, suggesting that teachers' confidence in managing dynamic sporting environments may be related to their creative teaching behavior.

A notable finding in our model was the strong positive association of Emotional Intelligence with both Wellbeing and Creative Teaching Behavior. This finding is consistent with Hargreaves's (1998) view that teaching is an emotional practice and with Yin et al. (2019), who highlighted the intense emotional labor required in physical education settings.

Our results suggest that EI may be related to teachers' capacity to manage emotional demands. According to Mérida-López and Extremera (2017), teachers with high EI are better at regulating negative emotions arising from student misbehavior or administrative pressure. High-EI teachers may be better positioned to manage emotional demands, which may leave more cognitive resources available for divergent thinking and creativity (Corcoran and Tormey, 2013). This pattern is consistent with Su et al. (2022), who suggested that in dynamic environments such as PE, the ability to perceive and manage emotions may be closely related to engagement. PE teachers with higher EI may be more likely to recognize student boredom or anxiety and to report adapting their instructional strategies creatively, a process Sawyer (2011) describes as “improvisational teaching.”

One contribution of this study is that it identified a significant statistical mediating role of Wellbeing. However, given the cross-sectional nature of the data, this mediating role should be interpreted as a statistical indirect association rather than evidence of a temporal or causal mechanism. While previous studies often treated wellbeing as a terminal outcome (Luthans et al., 2013; Siu et al., 2015), our model suggests that wellbeing is positively associated with creative teaching behavior and may be involved in an indirect pathway linking personal resources with CTB.

This finding provides empirical support for Fredrickson's (2001); Fredrickson (2004) Broaden-and-Build theory within the PE context. The positive emotions associated with wellbeing (e.g., feeling successful, connectedness) broaden the teachers' momentary thought-action repertoires. Unlike the “survival mode” triggered by stress (Hobfoll, 1989), a state of wellbeing encourages exploration and risk-taking. This may help explain why teachers in our sample who reported higher wellbeing also reported greater use of flexible and novel teaching methods.

Our measurement of wellbeing, which included “School Connectedness” and “Teaching Efficacy” (Renshaw et al., 2015), aligns with the eudaimonic perspective (Ryff, 1989; Waterman, 1993). It suggests that creativity is not just driven by hedonic happiness (Diener, 1984), but by a deeper sense of functioning and meaning at work. This nuances the findings of Xiong et al. (2020), who linked “work input” (vigor) to creativity. Our study suggests that teachers' psychological wellbeing may help explain the indirect association between personal resources and self-reported creative teaching behavior.

Integrating these findings, our study supports the “gain spiral” hypothesis of COR theory (Salanova et al., 2010; Hobfoll et al., 2018). PE teachers with higher levels of PsyCap and EI reported higher levels of wellbeing, which is consistent with the idea that personal resources may be associated with resource gain. This wellbeing was, in turn, positively associated with self-reported Creative Teaching Behavior.

This counters the “depletion” narrative often found in teacher burnout literature (Maslach et al., 2001; Madigan et al., 2023). Instead of viewing PE teaching solely as resource-draining, our data suggests that when equipped with the right psychological HERO (PsyCap) and relational tools (EI), teaching can be a regenerative process where wellbeing and creativity reinforce one another. This supports Bertieaux et al. (2024)'s recent assertion that focusing on positive psychological resources offers a more promising avenue for teacher retention and performance than merely mitigating stress.

The findings of this research may offer implications for higher education administration and teacher development by identifying psychological factors associated with teacher wellbeing and creative teaching behavior.

Traditional teacher training often focuses primarily on pedagogical content knowledge—what to teach and how to teach it. However, as evidenced by Lupşa et al. (2020), teachers' psychological resources play a critical role in their effectiveness and job satisfaction. The findings suggest that Psychological Capital may be a relevant consideration in teacher development. Although the present study did not test Psychological Capital Interventions, future programs may consider incorporating elements related to resilience, optimism, hope, and self-efficacy, as these resources were positively associated with wellbeing and Creative Teaching Behavior in the present sample. At the institutional level, universities may consider teacher development activities that attend not only to pedagogical skills but also to psychological resources. However, such suggestions should be treated as tentative and should be evaluated through future intervention or longitudinal research. By including these psychological resources in training, universities may help teachers develop a more adaptive and positive mindset toward their work, which may be associated with better wellbeing and classroom performance.

Emotional Intelligence (EI) has been shown to have a profound impact on teachers‘ ability to manage stress, engage with students, and maintain mental health (Vesely et al., 2013). Emotional labor, the management and regulation of one's emotions in a professional setting, is a significant aspect of teaching, especially in environments where interpersonal interactions are constant and emotionally charged. PE (Physical Education) teaching, in particular, involves a high level of emotional labor, as teachers need to manage not only their own emotions but also the emotional needs of students, especially in physical or competitive settings. Given the positive association between Emotional Intelligence and both wellbeing and Creative Teaching Behavior, universities may consider supporting teachers' emotional awareness and emotion-regulation capacities as part of professional development. Nevertheless, the present findings do not establish that EI training would cause improvements in wellbeing or creative teaching. For instance, they might offer courses on emotional regulation techniques, such as cognitive restructuring to handle classroom challenges, or mindfulness practices that help teachers stay present and manage their emotional responses effectively. Training could also include workshops on how to recognize and address emotional burnout, giving teachers the tools they need to maintain their mental health. Another practical implication is that universities may consider providing teachers with access to mental health resources, such as counseling services, to help them manage the emotional demands of teaching. These initiatives would be aligned with the recommendations of Vesely et al. (2013), who emphasize the importance of mental health support in maintaining teacher wellbeing.

Eudaimonic wellbeing refers to a deeper sense of wellbeing derived from meaningful engagement in life and work, rather than just the pursuit of happiness or pleasure. A key factor in fostering eudaimonic wellbeing is creating environments that support school connectedness, a sense of belonging and attachment to the institution that has been shown to positively affect both students and teachers (Renshaw et al., 2015). To support eudaimonic wellbeing, universities may consider creating a culture where teachers feel a strong sense of belonging to the academic community. This could involve promoting collaborative teaching initiatives, providing platforms for teachers to share their experiences and successes, and encouraging faculty participation in decision-making processes. For instance, a university could organize regular meetings where teachers from different departments collaborate on cross-disciplinary projects, fostering a sense of community and shared purpose. Additionally, administrators could support teacher engagement through recognition programs, where teachers are publicly acknowledged for their contributions to the academic and social environment of the university. Such programs would not only support teachers' wellbeing but also promote a culture of innovation, as teachers who feel supported and connected are more likely to contribute creative ideas and new approaches to their work.

## 6 Conclusion

This study explored the interactive relationship between Psychological Capital (PsyCap), Emotional Intelligence (EI), Wellbeing, and Creative Teaching Behavior (CTB) among Higher Education Physical Education (PE) teachers. The findings contribute to the literature by offering a comprehensive model that integrates the Conservation of Resources (COR) theory and Fredrickson's Broaden-and-Build theory within the context of PE teaching.

The results suggested that both PsyCap and EI were significantly associated with CTB, with wellbeing showing a significant statistical mediating role in these associations. Teachers with higher levels of PsyCap and EI reported greater wellbeing, which was also positively associated with self-reported creative teaching behavior. These findings suggest that creative teaching behavior is associated not only with personal resources such as resilience, optimism, and emotional regulation, but also with teachers' psychological state of wellbeing. Importantly, wellbeing was positively associated with creative teaching behavior and statistically mediated the associations between PsyCap, EI, and CTB. However, given the cross-sectional design, this result should not be interpreted as evidence that wellbeing is a temporal precursor to creative teaching.

These results have important practical implications for higher education institutions. The integration of Psychological Capital and Emotional Intelligence training into professional development programs for PE teachers could foster a more supportive environment for creativity in teaching. By strengthening the psychological resources of teachers, universities can enhance both teacher wellbeing and instructional quality. Furthermore, creating a culture that supports teacher wellbeing, emotional regulation, and psychological resilience may lead to more innovative and engaging physical education experiences for students.

However, this study has several limitations. First, the cross-sectional design of the study limits the ability to draw causal conclusions about the relationships among PsyCap, EI, wellbeing, and creative teaching behavior. Longitudinal studies would be valuable for examining the directionality and potential changes in these relationships over time. Second, the sampling strategy may limit the external validity of the findings. This study used convenience sampling supplemented by snowball sampling, which may have introduced selection bias. Teachers who were willing to participate in an online survey or who were connected to the researchers' professional networks may differ systematically from those who did not participate. For example, participants may have had stronger professional engagement, higher interest in psychological development, greater openness to creative teaching, or more supportive institutional environments. Therefore, the present sample should not be regarded as fully representative of all higher education PE teachers in Mainland China. In addition, the sample consisted exclusively of higher education PE teachers in Mainland China. The findings may not generalize directly to PE teachers in primary or secondary schools, teachers in other academic disciplines, or teachers working in different national and cultural contexts. Institutional expectations, teacher evaluation systems, professional autonomy, and cultural understandings of wellbeing and creativity may vary across educational systems. These contextual differences may shape the relationships among Psychological Capital, Emotional Intelligence, wellbeing, and Creative Teaching Behavior. Future research should use probability-based or stratified sampling strategies, include teachers from different regions and institutional types, and conduct cross-cultural comparisons to examine the robustness and boundary conditions of the present model. Future research could examine these constructs across different regions and educational systems. Third, while the self-report nature of the data collection is a common approach in educational research, it may be subject to response biases such as social desirability or common method bias. Incorporating objective measures of teaching behavior or peer evaluations could enhance the validity of the results. Finally, this study focused exclusively on PE teachers, and the findings may not be directly applicable to teachers in other disciplines. Expanding the research to include a wider range of teaching contexts could provide a broader understanding of how psychological resources influence creativity in teaching.

In conclusion, this study provides empirical evidence that PsyCap, EI, wellbeing, and creative teaching behavior are positively associated among higher education PE teachers. These findings suggest that supporting teachers' psychological resources and wellbeing may be relevant to fostering creative teaching, although causal conclusions cannot be drawn from the present cross-sectional data. Further research could explore how these psychological resources manifest in different educational contexts and how they evolve over time to sustain creativity in teaching practices.

## Data Availability

The original contributions presented in the study are included in the article/Supplementary material, further inquiries can be directed to the corresponding author.
