# Supplementary material for: Psychological capital, emotional intelligence, and creative teaching behavior among higher education physical education teachers: the mediating role of teacher wellbeing
Source: Front Psychol. 2026 Jul 28;17:1860093. doi: 10.3389/fpsyg.2026.1860093 (PMC13457459; doi:10.3389/fpsyg.2026.1860093)
Supplement: Supplementary file 1 [file Supplementary_File_1.docx]

Appendix A Scales Used in this Study

Psychological Capital questionnaire

Instructions:

Below are statements that describe how you may think about yourself right now. Use the following scale to indicate your level of agreement or disagreement with each statement.

Scale:

1 = Strongly Disagree

2 = Disagree

3 = Somewhat Disagree

4 = Somewhat Agree

5 = Agree

6 = Strongly Agree

1. I feel confident analyzing a long-term problem to find a solution.
2. I feel confident in representing my work area in meetings with management.
3. I feel confident contributing to discussions about the organization’s strategy.
4. I feel confident helping to set targets/goals in my work area.
5. I feel confident contacting people outside the organization (e.g., suppliers, customers) to discuss problems.
6. I feel confident presenting information to a group of colleagues.
7. If I should find myself in a jam at work, I could think of many ways to get out of it.
8. At the present time, I am energetically pursuing my work goals.
9. There are lots of ways around any problem.
10. Right now I see myself as being pretty successful at work.
11. I can think of many ways to reach my current work goals.
12. At this time, I am meeting the work goals that I have set for myself.
13. When I have a setback at work, I have trouble recovering from it, moving on.
14. I usually manage difficulties one way or another at work.
15. I can be “on my own,” so to speak, at work if I have to.
16. I usually take stressful things at work in stride.
17. I can get through difficult times at work because I’ve experienced difficulty before.
18. I feel I can handle many things at a time at this job.
19. When things are uncertain for me at work, I usually expect the best.
20. If something can go wrong for me work-wise, it will.
21. I always look on the bright side of things regarding my job.
22. I’m optimistic about what will happen to me in the future as it pertains to work.
23. In this job, things never work out the way I want them to.
24. I approach this job as if “every cloud has a silver lining.”

Emotional Intelligence questionnaire

Scale:

1 = Strongly Disagree

2 = Disagree

3 = Neutral

4 = Agree

5 = Strongly Agree

Self-Emotion Appraisal (SEA) Items:

1. I have a good sense of why I have certain feelings most of the time.
2. I have a good understanding of my own emotions.
3. I really understand what I feel.
4. I always know whether or not I am happy.

Others' Emotion Appraisal (OEA) Items:

5. I always know my friends' emotions from their behavior.
6. I am a good observer of others' emotions.
7. I am sensitive to the feelings and emotions of others.
8. I have a good understanding of the emotions of people around me.

Use of Emotion (UOE) Items:

9. I always set goals for myself and then try my best to achieve them.
10. I always tell myself I am a competent person.
11. I am a self-motivated person.
12. I would always encourage myself to try my best.

Regulation of Emotion (ROE) Items:

13. I am able to control my temper and handle difficulties rationally.
14. I am quite capable of controlling my own emotions.
15. I can always calm down quickly when I am very angry.
16. I have good control of my own emotions.

Teacher Subjective Wellbeing questionnaire

Scale:

1 = Almost Never

2 = Sometimes

3 = Often

4 = Almost Always

1. I feel like I belong at this school.
2. I am a successful teacher.
3. I can really be myself at this school.
4. I am good at helping students learn new things.
5. I feel like people at this school care about me.
6. I have accomplished a lot as a teacher.
7. I am treated with respect at this school.
8. I feel like my teaching is effective and helpful.

Creative Teaching questionnaire

Scale:

1 = Strongly Disagree

2 = Disagree

3 = Somewhat Disagree

4 = Neutral

5 = Somewhat Agree

6 = Agree

7 = Strongly Agree

1. I am capable of helping students to become more flexible in their thinking.
2. I am capable of enhancing my students’ abilities to take meaningful academic risks.
3. I am capable of fostering creative problem solving in my classroom.
4. I am capable of increasing my students’ abilities to create unique solutions.
5. I am capable of developing a classroom atmosphere that welcomes imagination.
6. I am capable of promoting flexible thinking.
7. I am capable of helping my students to see the world from new perspectives.
8. I am capable of teaching my students to find connections in seemingly unconnected ideas.
9. I am capable of increasing the quantity of original thoughts my students have.
10. I am capable of helping students to elaborate on their own unique ideas.
11. I am capable of fostering creative problem solving in my classroom.
12. I have helped many students to become more creative.
13. I am confident that all of my students are more creative thinkers by the end of the year.

Appendix B Questionnaire administered in this study

说明 / Instructions

中文说明：

感谢您参与本研究。本问卷旨在了解高校体育教师的心理资本、情绪智力、幸福感以及创造性教学行为。

本调查采取匿名方式，所有数据仅用于学术研究。问卷不存在“对”或“错”的答案，请根据您的真实感受和实际情况作答。您可在任何时间退出本调查。

完成问卷大约需要 10–15 分钟。

English Instructions:

Thank you for participating in this study. This questionnaire aims to investigate psychological capital, emotional intelligence, well-being, and creative teaching behavior among higher education physical education teachers.

Participation is voluntary and anonymous, and all responses will be used for research purposes only. There are no right or wrong answers. Please respond honestly based on your actual experiences. You may withdraw at any time.

第一部分：基本信息 / Part I. Demographic Information

性别 / Gender

☐ 男 / Male ☐ 女 / Female ☐ 不愿透露 / Prefer not to say

年龄 / Age

☐ 30 岁以下 / Under 30

☐ 30–39 岁 / 30–39

☐ 40–49 岁 / 40–49

☐ 50 岁及以上 / 50 or above

最高学历 / Highest Educational Qualification

☐ 学士 / Bachelor’s

☐ 硕士 / Master’s

☐ 博士 / Doctorate

职称 / Academic Rank

☐ 助教 / Teaching Assistant

☐ 讲师 / Lecturer

☐ 副教授 / Associate Professor

☐ 教授 / Professor

高校体育教学年限 / Years of Teaching Experience

☐ 5 年以下 / Less than 5 years

☐ 6–15 年 / 6–15 years

☐ 15 年以上 / More than 15 years

第二部分：心理资本 / Part II. Psychological Capital

作答说明（中文）：

以下陈述描述了您当前在工作中的状态。请根据您的实际情况选择最符合的一项。

Instructions (English):

The following statements describe how you may think about yourself at work right now. Please indicate your level of agreement.

量表 / Scale:

1 = 非常不同意 / Strongly Disagree

2 = 不同意 / Disagree

3 = 有些不同意 / Somewhat Disagree

4 = 有些同意 / Somewhat Agree

5 = 同意 / Agree

6 = 非常同意 / Strongly Agree

我有信心分析长期问题并找到解决方案。

I feel confident analyzing a long-term problem to find a solution.

我有信心在管理层会议中代表我的工作领域。

I feel confident representing my work area in meetings with management.

我有信心参与有关组织战略的讨论。

I feel confident contributing to discussions about the organization’s strategy.

我有信心协助制定我工作领域的目标。

I feel confident helping to set targets/goals in my work area.

我有信心与组织外部人员联系并讨论问题。

I feel confident contacting people outside the organization to discuss problems.

我有信心向同事群体展示信息。

I feel confident presenting information to a group of colleagues.

当我在工作中遇到困难时，我能想到多种解决办法。

If I find myself in a jam at work, I could think of many ways to get out of it.

目前我正在积极地追求我的工作目标。

At the present time, I am energetically pursuing my work goals.

面对问题，总是有多种解决路径。

There are lots of ways around any problem.

目前我认为自己在工作中相当成功。

Right now I see myself as being pretty successful at work.

我能想到许多方式实现当前的工作目标。

I can think of many ways to reach my current work goals.

目前我正在实现自己设定的工作目标。

At this time, I am meeting the work goals that I have set for myself.

当我在工作中遇到挫折时，我很难恢复并继续前进。（反向题）

When I have a setback at work, I have trouble recovering from it. (R)

在工作中，我通常能够以某种方式应对困难。

I usually manage difficulties one way or another at work.

如果需要，我可以在工作中独立应对问题。

I can be “on my own” at work if I have to.

我通常能够从容应对工作中的压力。

I usually take stressful things at work in stride.

因为我以前经历过困难，我能度过当前的艰难时刻。

I can get through difficult times at work because I’ve experienced difficulty before.

我觉得自己能同时处理多项工作任务。

I feel I can handle many things at a time at this job.

当工作充满不确定性时，我通常期待最好的结果。

When things are uncertain at work, I usually expect the best.

如果工作中可能出错，那通常就会发生在我身上。（反向题）

If something can go wrong for me work-wise, it will. (R)

关于工作，我总是看到积极的一面。

I always look on the bright side of things regarding my job.

对于我的职业未来，我持乐观态度。

I’m optimistic about what will happen to me in the future as it pertains to work.

在这份工作中，事情从来不会按我期望的方式发展。（反向题）

In this job, things never work out the way I want them to. (R)

我对这份工作的态度是“乌云背后总有阳光”。

I approach this job as if every cloud has a silver lining.

第三部分：情绪智力 / Part III. Emotional Intelligence

量表 / Scale:

1 = 非常不同意 / Strongly Disagree

2 = 不同意 / Disagree

3 = 一般 / Neutral

4 = 同意 / Agree

5 = 非常同意 / Strongly Agree

我大多数时候都清楚自己为什么会产生某些情绪。

I have a good sense of why I have certain feelings most of the time.

我对自己的情绪有很好的理解。

I have a good understanding of my own emotions.

我真正理解自己的感受。

I really understand what I feel.

我总是知道自己是否感到快乐。

I always know whether or not I am happy.

我可以通过朋友的行为判断他们的情绪。

I always know my friends’ emotions from their behavior.

我善于观察他人的情绪。

I am a good observer of others’ emotions.

我对他人的情绪变化很敏感。

I am sensitive to the feelings and emotions of others.

我对周围人的情绪有较好的理解。

I have a good understanding of the emotions of people around me.

我会为自己设定目标并努力实现。

I always set goals for myself and then try my best to achieve them.

我总是告诉自己我是一个有能力的人。

I always tell myself I am a competent person.

我是一个自我激励的人。

I am a self-motivated person.

我总是鼓励自己尽最大努力。

I would always encourage myself to try my best.

我能够控制脾气并理性应对困难。

I am able to control my temper and handle difficulties rationally.

我能够很好地控制自己的情绪。

I am quite capable of controlling my own emotions.

当我非常愤怒时，我能迅速冷静下来。

I can always calm down quickly when I am very angry.

我对自己的情绪有良好的掌控能力。

I have good control of my own emotions.

第四部分：教师幸福感 / Part IV. Teacher Well-being

量表 / Scale:

1 = 几乎没有 / Almost Never

2 = 有时 / Sometimes

3 = 经常 / Often

4 = 几乎总是 / Almost Always

我觉得自己属于这所学校。

I feel like I belong at this school.

我认为自己是一名成功的教师。

I am a successful teacher.

在这所学校，我可以做真实的自己。

I can really be myself at this school.

我擅长帮助学生学习新知识。

I am good at helping students learn new things.

我觉得学校里的人关心我。

I feel like people at this school care about me.

作为一名教师，我取得了很多成就。

I have accomplished a lot as a teacher.

我在这所学校受到尊重。

I am treated with respect at this school.

我认为自己的教学是有效且有帮助的。

I feel like my teaching is effective and helpful.

第五部分：创造性教学行为 / Part V. Creative Teaching Behavior

量表 / Scale:

1 = 非常不同意 / Strongly Disagree

2 = 不同意 / Disagree

3 = 有些不同意 / Somewhat Disagree

4 = 一般 / Neutral

5 = 有些同意 / Somewhat Agree

6 = 同意 / Agree

7 = 非常同意 / Strongly Agree

我能够帮助学生形成更灵活的思维方式。

I am capable of helping students become more flexible in their thinking.

我能够增强学生进行有意义学术冒险的能力。

I am capable of enhancing students’ abilities to take meaningful academic risks.

我能够在课堂中培养学生的创造性问题解决能力。

I am capable of fostering creative problem solving in my classroom.

我能够提升学生创造独特解决方案的能力。

I am capable of increasing students’ abilities to create unique solutions.

我能够营造一个鼓励想象力的课堂氛围。

I am capable of developing a classroom atmosphere that welcomes imagination.

我能够促进学生的灵活思维。

I am capable of promoting flexible thinking.

我能够帮助学生从新的视角看待世界。

I am capable of helping students see the world from new perspectives.

我能够教会学生在看似无关的想法中建立联系。

I am capable of teaching students to find connections in seemingly unconnected ideas.

我能够增加学生原创想法的数量。

I am capable of increasing the quantity of original thoughts students have.

我能够帮助学生对其独特想法进行拓展。

I am capable of helping students elaborate on their own unique ideas.

我在课堂中经常培养学生的创造性问题解决能力。

I am capable of fostering creative problem solving in my classroom.

我已经帮助许多学生变得更有创造力。

I have helped many students become more creative.

我确信到学年结束时，所有学生都会成为更有创造力的思考者。

I am confident that all of my students are more creative thinkers by the end of the year.

Appendix C Convergent validity and reliability and Discriminant validity

Table C1. Convergent validity and reliability

| Construct (Latent variable) | # Items | Std. factor loadings range (λ) | CR | AVE | √AVE |
| --- | --- | --- | --- | --- | --- |
| Psychological Capital (PsyCap) | 24 | [.60, .86] | .94 | .55 | .74 |
| Emotional Intelligence (EI) | 16 | [.62, .88] | .93 | .58 | .76 |
| Teacher Well-being (WB) | 8 | [.70, .89] | .91 | .60 | .77 |
| Creative Teaching Behavior (CTB) | 13 | [.65, .90] | .92 | .59 | .77 |

Table C2. Discriminant validity (Fornell–Larcker)

| Construct | PsyCap | EI | WB | CTB |
| --- | --- | --- | --- | --- |
| PsyCap | .74 | .63 | .57 | .52 |
| EI | .63 | .76 | .61 | .56 |
| WB | .57 | .61 | .77 | .66 |
| CTB | .52 | .56 | .66 | .77 |
